# Profile of cancer patients’ seen at Korle Bu teaching hospital in Ghana (A cancer registry review)

**DOI:** 10.1186/1756-0500-7-577

**Published:** 2014-08-27

**Authors:** Benedict NL Calys-Tagoe, Joel Yarney, Ernest Kenu, Nana Adwoa K Owusu Amanhyia, Ernest Enchill, Isaac Obeng

**Affiliations:** Public Health Unit, Korle-Bu Teaching Hospital, Korle-Bu, Accra, Ghana; Department of Community Health, University of Ghana Medical School, College of Health Sciences, Korle-Bu, Accra, Ghana; National Centre for Radiotherapy and Nuclear Medicine, Korle Bu, Accra, Ghana; School of Public Health, University of Ghana, Accra, Ghana

**Keywords:** Profile, Cancers, Korle Bu teaching hospital, Ghana

## Abstract

**Background:**

Though cancer has become a major source of morbidity and mortality globally, few countries in Sub Saharan Africa have data on cancer incidence. This study aims to describe the profile of cancers seen at the Korle Bu teaching hospital which is a major referral centre in Ghana for cancers and other health conditions.

Data for the study was obtained from the cancer registry of the hospital and covered the period from January 2012 to December 2012. The public health unit actively collects data on all cancer cases presenting to any department/unit of the hospital to feed the cancer registry.

**Results:**

A total of 1136 patients with cancer were studied. Their ages ranged from 1 year to 92 years with a mean of 52.3 ± 15.9 years and a median of 54 years. Patients were predominantly female (70.2%) and majority had attained secondary level of education or higher. The most prevalent cancers seen in men were those of prostate, pharynx and colorectal while in the females, the corresponding cancers were breast, cervix and uterus.

**Conclusions:**

Breast and prostate cancers were the commonest among females and males respectively who presented with cancer at the Korle Bu teaching hospital in 2012.

## Background

Cancer continues to be a leading cause of death in many economically developed countries as well as the second leading cause of death in developing countries [1]. This occurrence may be attributed to aging of the world populations due to improving life expectancies and the adaptation of “cancer-causing” lifestyle and behaviors such as smoking, physical inactivity and the uptake of junk and/or fast foods which are high in fats, oils and salt. It may also be due to improved ways of diagnosing cancers as well as the establishment of cancer registries in many countries worldwide [2].

There were 14.1 million new cancer cases, 8.2 million cancer deaths and 32.6 million people living with cancer (within 5 years of diagnosis) in 2012 worldwide, 57% (8 million) of new cancer cases, 65% (5.3 million) of the cancer deaths and 48% (15.6 million) of the 5-year prevalent cancer cases occurred in the less developed regions [3].

Although there are several types of cancers some of which cuts across both sexes, there are others that are more common within a particular sex. Breast cancer in females and lung cancer in males are the most frequently diagnosed cancers and the leading cause of cancer death for each sex worldwide. However lung cancer is preceded by prostate cancer as the most frequent cancer among males in economically developed countries [4]. These cancers were followed in no specific order by stomach and liver cancers in males and cervix and lung cancers in females in economically developing countries and by colorectal and lung cancers in females and colorectal and lung or prostate cancers in males in the economically developed worlds [4].

Cancers of the lung, stomach, colon and rectum, liver, and esophagus are associated with high incidence worldwide. The same can be said of sex-specific malignancies of the female breast, uterine cervix, and prostate [5]. Whereas cancers of the female breast and prostate are among the most common cancers worldwide, mortality rates are comparatively low, consistent with favorable survival. In contrast, cancers of the lung, liver, and esophagus are associated with high mortality – mortality rate: incidence rate (MR: IR) approaching 1.00, indicative of poor survival [5].

Though cancer has become a major source of morbidity and mortality globally, only few countries in Sub Saharan Africa have data on cancer incidence. According to GLOBOCAN 2012, the commonest cancers among males in Ghana are liver cancer, prostate cancer and non-hodgkin lymphoma while that for females are; cervical cancer, breast cancer and liver cancer in decreasing order of prevalence [3]. In The Gambia, the commonest cancers according to Sighoko et al, are liver cancer, prostate cancer and nonhodgkin lymphoma for males and cervical cancer, liver cancer and breast cancer for females [6]. A study of patients with advance cancers at Lukas clinic in Switzerland revealed that the patients were primarily female, had higher education and lived in urban environment [7]. In another study carried out in Nigeria in 2012, 66% of the cancer patients were female with the remaining 34% being male. While breast and cervical cancer were the commonest cancers among women, prostate cancer was the most common among men [8].

The profile of cancer patients seen at Korle-Bu teaching hospital (KBTH) has not been documented since the establishment of the cancer registry at the beginning of 2012. With that in mind, this study aimed to describe the profile of cancers seen at the Korle Bu teaching hospital which is a major referral centre for cancers and other health conditions and also to highlight areas that require improvement to improve the quality and completeness of data from the hospital cancer registry.

## Methods

### Study location

The study was carried out at the Korle Bu Teaching Hospital (KBTH) in Accra, Ghana. The Korle Bu Teaching Hospital is the largest health facility, premier teaching hospital and nerve centre of healthcare services in Ghana. It serves as the main referral center for the entire southern Ghana and beyond. It currently has a bed capacity of over 2000 and serves as a training center for medical doctors, nurses and other health professionals. The hospital has 17 clinical and diagnostic departments/units including three centers of excellence namely; The National Cardiothoracic Centre, The Reconstructive Plastic Surgery and Burns Centre and the National Centre for Radiotherapy and Nuclear Medicine which serves as a major referral centre for the management of cancers, with clients extending beyond the borders of Ghana to neighboring Nigeria, Burkina Faso and Togo. Many specialized services including renal transplantation, DNA investigations and brachy therapy for the treatment of prostate cancer are offered by the hospital. It was also the first hospital in Ghana to carry out ureteroscopy [9]. With respect to cancer management, there are very few facilities besides KBTH in Accra with capacity to diagnose and/or effectively manage cases.

Korle-Bu Teaching Hospital gave approval for the establishment of the cancer registry and carry out review of the system. Data for the study was obtained from the cancer registry of the hospital (which was established at the beginning of 2012) and covered the period from January 2012 to December 2012. The cancer registry actively collects data on all cancer cases presenting to all departments/units of the hospital. This involves regular visits to the various clinics and wards of admission as well as laboratories by staff of the registry (Public health nurses) to abstract the required information from the records (folders) of patients using a specially designed abstraction form with succinct case definition.

### Population covered

This is a hospital based registry with a wide catchment covering the entire southern sector of the country and beyond and therefore has no well-defined population. However, the total attendance for the year under review (413,514) was used as the denominator for calculations.

### Data handling

Data collected was captured using the CanReg 5 software designed by the International Agency for Research into Cancer (IARC), which has an in-built mechanism for detecting duplication. This ensured that information on the same patient was not captured more than once. Additionally, the registration numbers of 10% of the records were randomly selected, their medical records retrieved and re-abstraction done by the research team. Minor errors, mainly typographical, which could be attributed to poor hand writing were identified. The identified errors were corrected before the data was used for this analysis. The data was exported into Microsoft Excel and SPSS version 16 for windows where the analyses were carried out.

## Results

**Figure 1:**
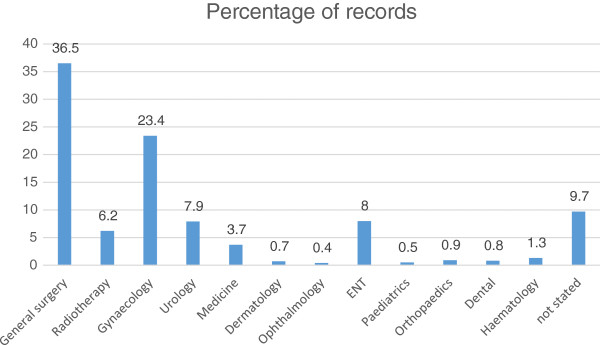
**Distribution of the cases by department/unit of the hospital.**

A total of 1136 patients with cancer were captured by the Korle Bu teaching hospital cancer registry between January and December 2012 out of a total hospital attendance of 413514. This gives a cancer prevalence of 274.7 cancers per 100,000 hospital attendees. Of these 1136 cases, 807 (71%) were diagnosed between the period January to December 2012. The 1136 cases were made up of 339 (29.8%) males and 797 (70.2%) females. Their ages ranged from 1 year to 92 years with a mean of 52.3 ± 15.9 years and a median of 54 years. The socio demographic characteristics of the patients are as shown in Table 1. The patients were mostly Ghanaians (88.1%), but some were from other African countries namely; Benin (2.1%), Burkina Faso (1.4%), Cote D’Ivoire (1.4%), The Gambia (0.1%), Guinea (0.1%), Liberia (0.8%), Libya (0.1%), Niger (0.1%), Nigeria (0.7%), Sierra Leone (1.5%) and Togo (3.6%). Seven hundred and seventy two (68%) of the records had no documentation of educational attainment. Of the 364 that had educational attainment documented, 232 (63.7%) had completed tertiary education, 52 (14.3%) had completed secondary education, 55 (15.1%) had primary education and 19 (5.2%) had no formal education- these included six children who were not of school going age. Overall, 704 (62%) of the cancer patients were married with the proportion of married men being much higher (77.6%) than the corresponding figure for women (55.3%). The distribution of the cases by departments/units of the hospital is shown in Figure 1.

**Table 1:** **Socio-demographic characteristics of patients**

Characteristic	Male N = 339 (%)	Female N = 797 (%)	Total N = 1136 (%)
**Age** **(years)**			
0-9	7 (2.1)	5 (0.6)	12 (1.1)
10-19	12 (3.5)	16 (2.0)	28 (2.5)
20-29	27 (8.0)	27 (3.4)	54 (4.8)
30-39	25 (7.4)	110 (13.8)	135 (11.9)
40-49	38 (11.2)	178 (22.3)	216 (19.0)
50-59	75 (22.1)	226 (28.5)	301 (26.4)
60-69	93 (27.5)	146 (18.3)	239 (21.0)
70-79	52 (15.3)	72 (9.0)	124 (10.9)
80-89	10 (2.9)	16 (2.0)	26 (2.3)
90-99	0 (0)	1 (0.1)	1 (0.1)
**Marital status**			
Married	263 (77.5)	441 (55.4)	704 (62.0)
Divorced	6 (1.8)	49 (6.1)	55 (4.8)
Single	56 (16.5)	224 (28.1)	280 (24.6)
Widowed	9 (2.7)	68 (8.5)	77 (6.8)
Missing	5 (1.5)	15 (1.9)	20 (1.8)
**Educational level**			
Tertiary	123 (36.2)	109 (13.7)	232 (20.4)
Secondary	22 (6.5)	30 (3.8)	52 (4.6)
Primary	18 (5.3)	37 (4.6)	55 (4.8)
None	4 (1.2)	15 (1.9)	19 (1.7)
Not applicable	4 (1.2)	2 (0.3)	6 (0.5)
Missing	168 (49.6)	604 (75.7)	772 (68.0)
**Religion**			
Christianity	297 (87.8)	683 (85.6)	980 (86.3)
Islam	35 (10.4)	87 (10.9)	122 (10.7)
Buddhism	1 (0.3)	0 (0)	1 (0.1)
Hinduism	0 (0)	1 (0.1)	1 (0.1)
ATR	4 (1.2)	1 (0.1)	5 (0.4)
None	1 (0.3)	26 (3.3)	27 (2.4)
**Ethnicity**			
Akan	142 (41.8)	297 (37.3)	439 (38.6)
Ga/Adangbe	65 (19.2)	150 (18.8)	215 (18.9)
Ewe	49 (14.5)	143 (17.9)	192 (16.9)
Northern dialects	27 (8)	55 (6.9)	82 (7.2)
Non-Ghanaians	35 (10.3)	102 (12.8)	137 (12.1)
Other Ghanaian dialects	17 (5.0)	44 (5.5)	61 (5.4)
Missing	4 (1.2)	6 (0.8)	10 (0.9)

### Anatomical sites of cancers seen

Breast cancer was the most common cancer seen at the Korle Bu teaching hospital in 2012. A total of 333 cases (making up 29.3% of all the cancers) were seen of which 198 were newly diagnosed. This gives a prevalence of 80.5 cases per 100,000 hospital attendees. Eight out of the 333 breast cancer cases occurred in males giving a prevalence of 1.9 cases per 100,000 cases seen for breast cancer in males. The other major cancers were cervical cancer 194 (46.9 cases per 100,000), prostate cancer 90 (21.8 cases per 100,000) and colorectal cancers 57 (13.8 cases per 100,000). The mean ages at diagnosis for these cancers were; 49.8 ± 12.5 for breast cancer, 57.4 ± 11.9 years for cervical cancer, 66.2 ± 7.1 years for prostate cancer and 54.6 ± 11.0 years for colorectal cancers.

Among the females, the common cancers were those of the breast (40.8%), cervix (24.3%) and uterus (4.5%). For the males, common cancers were those of the prostate (26.5%), pharynx (7.4%) and colorectal (6.5%). Tables 2 and 3 show the ten most prevalent cancers and their incidence rates.

**Table 2:** **Ten most prevalent cancers seen at KBTH by sexes in 2012**

	Overall		Male		Female	
No.	Site	N (%)	Site	N (%)	Site	N (%)
1.	Breast	333 (29.3)	Prostate	90 (26.5)	Breast	325 (40.8)
2.	Cervix	194 (17.1)	Pharynx	25 (7.4)	Cervix	194 (24.3)
3.	Prostate	90 (7.9)	Colorectal	22 (6.5)	Uterus	36 (4.5)
4.	Colorectal	57 (5.0)	Stomach	19 (5.6)	Colorectal	35 (4.4)
5.	Uterus	36 (3.2)	Bones	17 (5.0)	Ovary	34 (4.3)
6.	Ovary	34 (3.0)	Skin	17 (5.0)	Thyroid	17 (2.1)
7.	Pharynx	33 (2.9)	Larynx	12 (3.5)	Bones	15 (1.9)
8.	Bones	32 (2.8)	Lung	9 (2.7)	Lung	10 (1.3)
9.	Skin	26 (2.3)	Liver	9 (2.7)	Skin	9 (1.1)
10.	Stomach	25 (2.2)	Bone marrow	8 (2.4)	Brain	9 (1.1)

**Table 3:** **Prevalence and incidence of most common cancers seen at KBTH in 2012**

Site	Total cases	^*^New cases	^**^Prevalence/100,000	^**^Incidence/100,000/year
Breast	333	198	80.5	47.9
Cervix	194	154	46.9	37.2
Prostate	90	54	21.8	13.1
Colorectal	57	48	13.8	11.6
Uterus	36	31	8.7	7.5
Ovary	34	29	8.2	7.0
Pharynx	33	25	8.0	6.0
Bones	32	24	7.7	5.8
Skin	26	19	6.3	4.6
Stomach	25	19	6.0	4.6

^*^New cases imply those first diagnosed between January and December 2012.

^**^The denominator for the calculation is 413,514.

### Basis of diagnosis

Out of the 1136 cases, 1027 (90.4%) were initially diagnosed based on the histology of the primary lesion, 23 (2.0%) were based on histology of the metastatic lesions and 21 (1.8%) were on the basis of cytology. Fifty six (4.9%) of the diagnosis were on the basis of clinical investigations such as various laboratory tests and ultrasonography. Only 7 (0.6%) of the diagnosis were based on clinical assessment only.

### Extent of disease at presentation

Only 303 (26.7%) out of the 1136 cases had this parameter documented in their hospital records. Of this number, 155 (51.1%) had localized disease, 40 (13.2%) had diseases that had extended into adjoining structures, 36 (11.9%) had regional lymph node involvement and 72 (23.8%) had distant metastases at the time of presentation.

## Discussion

The study involved 1136 cases of cancer captured by the cancer registry of the Korle Bu teaching hospital. Of these 797, constituting 70.2% were females. This is comparable to the finding of previous studies [7, 8, 10]. This could be attributed to the fact that three out of the five most prevalence cancers seen-breast, cervix and uterus-occur predominantly, if not exclusively in females.

The mean age of diagnosis of 52.3 ± 15.9 years obtained in this study is similar to that observed in another study conducted in Pakistan in 2012 where the mean age of cancer patients was 51.8 ± 14.2 years [11], but higher than that observed for men and women in Abuja (49.9 years and 45.4 years respectively) [8].

Close to 70% of the records did not contain information on educational attainment while over 70% had no documentation on the extent of disease at presentation. This is very unsatisfactory and we recommend that hospital management educate staff of the records department and clinicians on the need to capture these variables and monitor them regularly to ensure that they are captured subsequently to improve data quality and completeness. Of the 364 cases that had their educational attainment recorded, 284, constituting 78% had attained secondary education or higher. This is also consistent with the finding of Pampollona et al, [7] in Switzerland where most of the patients who reported to the hospital had higher education. Level of education is known to influence health seeking behavior with the more educated being more likely to seek health. This finding is therefore not surprising as the data in both situations were obtained from health facilities.

Breast cancer was found to be the most prevalent cancer in this study accounting for nearly a third (29.3%) of all cancers seen as was also found by Bhurgri et al, in Karachi, Pakistan [11]. However, Biritwum et al, [12] in their study of patterns of diseases leading to hospitalization at Korle Bu teaching hospital in 1996, indicated Burkitt’s tumour as the leading malignancy requiring admission. This was followed by breast cancer and malignancies of the urinary tract. Most cases of breast cancer are managed as outpatients (not requiring admission) except when they require surgical intervention and that could account for the difference in findings as they focused on admitted cases. Breast cancer and cervical cancer were the most prevalent cancers among women while prostate cancer was the commonest cancer seen among men at the Korle Bu teaching hospital in 2012. This is similar to what was reported from Nigeria [8] which is also a developing country like Ghana. In this study, the three commonest cancers among men were found to be that of prostate (21.8 per 100,000), pharynx (6 per 100,000) and colorectal (5.3 per 100,000). In the United States, the three most common cancers among men were prostate, lung and colorectal cancers [13]. Sighoko et al. [6], in their evidence from 19 years of population-based cancer registration in The Gambia, however, found liver cancer to be the most common among men. It was followed by prostate and non-Hodgkin lymphoma. In women, they found cervical cancer to be the commonest followed by liver and breast cancer. Their findings are quite similar to that contained in GLOBOCAN 2012 for Ghana [3].

Contrary to the results of other studies [3–6]; the prevalence and incidence of lung as well as liver cancers in this study were low. The low prevalence could be attributable to the high mortality rate: incident rate ratios associated with these malignancies and the low incidence levels. The low incidence however could be the combined effect of “missed diagnosis” and the failure of the system to capture these malignancies even when they are diagnosed. The clinics where these malignancies are most likely to be captured are run in the afternoons. Due to the inadequate numbers of public health staff there is either few or none at all to capture data during the afternoon clinics, contributing to the low incidence levels.

Over 94% (1071) of the cases were initially diagnosed on the basis of histological or cytological tests with only 0.6% being diagnosed on clinical assessment only. This relatively low proportion of cases diagnosed on clinical grounds alone coupled with the high proportion of histological/cytological diagnosed cases may suggest a probable under-reporting of cases [14].

The results of this study have been presented to the management board of the Korle Bu Teaching Hospital and it is the hope of the authors that the findings will inform its decisions to improve the operations of the hospital cancer registry.

### Study limitation

This study depended on existing data collected by public health staff for the hospital registry. The public health staff were challenged in terms of numbers and therefore may not have been able to completely capture data from all outpatient clinics within the hospital. This may lead to under reporting of some malignancies. Therefore the actual numbers may be much higher than stated in this study. Secondly, rates presented in this study were generated using total hospital attendance (registry is hospital-based) and not the general population and therefore they should be interpreted with that in mind.

## Conclusions

Cancers of the breast, cervix and uterus were the commonest cancers found among females while cancer of the prostate, pharynx and colorectal region were the commonest among males reporting with cancer at the Korle Bu teaching hospital in 2012.
